# A Genome-Wide Analysis and Expression Profile of Heat Shock Transcription Factor (Hsf) Gene Family in *Rhododendron simsii*

**DOI:** 10.3390/plants12223917

**Published:** 2023-11-20

**Authors:** Yanan Xu, Ying Jin, Dan He, Haochen Di, Ying Liang, Yanxia Xu

**Affiliations:** 1 Jiyang College, Zhejiang A&F University, Zhuji 311800, China; 18314859885@163.com (Y.X.); dhc200528@163.com (H.D.); liangy202303@163.com (Y.L.); 2 College of Advanced Agricultural Sciences, Zhejiang A&F University, Hangzhou 311300, China; 3 Zhuji Economic Specialty Station, Zhuji 311800, China; 13606560006@163.com (Y.J.); hedan821022@163.com (D.H.)

**Keywords:** *Rhododendron*, RsHsf family, abiotic stress, gene expression

## Abstract

Heat shock transcription factors are key players in a number of transcriptional regulatory pathways that function during plant growth and development. However, their mode of action in *Rhododendron simsii* is still unclear. In this study, 22 *RsHsf* genes were identified from genomic data of *R. simsii*. The 22 genes were randomly distributed on 12 chromosomes, and were divided into three major groups according to their phylogenetic relationships. The structures and conserved motifs were predicted for the 22 genes. Analysis of cis-acting elements revealed stress-responsive and phytohormone-responsive elements in the gene promoter regions, but the types and number varied among the different groups of genes. Transcriptional profile analyses revealed that *RsHsfs* were expressed in a tissue-specific manner, with particularly high transcript levels in the roots. The transcriptional profiles under abiotic stress were detected by qRT-PCR, and the results further validated the critical function of *RsHsfs*. This study provides basic information about RsHsf family in *R. simsii*, and paves the way for further research to clarify their precise roles and to breed new stress-tolerant varieties.

## 1. Introduction

With global climate change, excessive heat is becoming one of the main environmental factors restricting plant growth. Transient or persistent heat stress negatively affects plant growth, and can result in death in severe cases. However, plants can detect even slight changes in temperature and respond accordingly. Plants have evolved a series of complex and efficient response mechanisms to adjust their morphology and physiology to adapt to environmental conditions [1,2]. Studies on the regulation mechanisms of plants have shown that heat shock transcription factors (Hsfs) are key transcription factors in the heat response and in signal transduction [3].

Hsfs were first discovered in yeast [4], and were subsequently found in *Drosophila* [5] and mammals [6,7]. Plant Hsfs were first discovered and cloned in tomato [4]. Then, 21 members of the Hsf family were identified from *Arabidopsis thaliana* [8,9] and classified according to their structural characteristics. This became the basis of Hsf research in the following decade. Other studies have since identified members of the Hsf family in many higher plants, including maize [10], cotton [11] and rice [12]. As a transcription factor, Hsf can combine with the heat shock element (HSE) and activate the expression of downstream genes during the response to heat stress [13,14,15].

Hsfs play crucial roles in plant growth and stress response, and their expression is induced by various stresses. For instance, *HsfA2* is a heat stress-inducible gene and its product regulates the expression of a subset of downstream stress response genes [16]. Overexpression of *HsfA2s* from *A. thaliana*, *Zea mays*, and *Oryza sativa* conferred heat tolerance in transgenic Arabidopsis [17,18,19]. In wheat, *TaHsfA6f* encodes a transcription factor that regulates the expression of several target genes (*TaHsps*, *TaRof1*, *TaGST*) to improve heat resistance [20]. In addition, *AtHsfA1s* were found to be involved in responses to other stresses, such as salt, osmotic, and oxidative stresses [21]. Overexpression of *CarHsfB2* led to increased expression levels of some stress-related genes (*RD22*, *RD26* and *RD29A*) in transgenic Arabidopsis under drought stress, and improved its drought tolerance [22]. However, *SlHsfA3* and *VpHsf1* were found to play a negative regulatory role in salt stress and osmotic stress, different from the positive roles of the regulatory factors mentioned above [23,24]. The results of those studies show that Hsf transcription factors play functionally diverse roles in stress responses.

*Rhododendron* is well known for its beautiful, brightly colored flowers, and is cultivated worldwide. In addition to their ornamental value, *Rhododendron* species have other uses in food and medicine [25]. For example, its root extracts have been shown to promote blood circulation, stop bleeding, reduce heat, and eliminate toxic materials. Essential oils are extracted from some cultivars. *Rhododendron* grows best in cool, humid, and ventilated semi-shady environments. The optimum temperature range for growth is 12 °C to 25 °C. When the temperature is too high, new sprouts and leaves grow slowly and become semi-dormant. Therefore, it is essential to study how the heat stress response is regulated in *Rhododendron*.

The Hsf gene family has been studied in many species, but has not been analyzed in detail in *Rhododendron*. In this study, 22 *RsHsfs* were identified, and their gene structure and phylogenetic relationships were determined. The physicochemical properties of the putative proteins and their conserved domains were predicted. The gene promoter regions were analyzed to detect cis-acting elements. The transcriptional profiles of *RsHsf* genes in different tissues and in response to various abiotic stresses were determined by qRT-PCR, which identified candidate genes involved in stress responses. The results of this study provide a theoretical reference for further studies on the Hsfs of *R. simsii* and their roles in heat tolerance.

## 2. Results

### 2.1. Gene Identification and Physicochemical Properties of Putative Proteins

BLASTX searches were performed using *AtHsf* gene sequence as queries. PFAM and SMART were used to remove the invalid and repetitive amino acid sequences obtained from *Rhododendron simsii* genome database [26]. In total, 22 *RsHsf* genes were identified and named *RsHsf1*–*RsHsf22* (Table 1). The proteins encoded by RsHsfs contained 140 (RsHsf2) to 740 (RsHsf3) amino acids (a.a.), with predicted molecular weights ranging from 16.63 (RsHsf2) to 83.56 (RsHsf3) KDa, and isoelectric points ranging from 4.5 (RsHsf2) to 9.13 (RsHsf22). All of the RsHsfs except for RsHsf16 and RsHsf19 were predicted to be unstable proteins. The grand average of hydropathicity values of RsHsfs were all negative. Therefore, RsHsfs were predicted to be predominantly hydrophilic proteins.

### 2.2. Location of Genes on Chromosomes

**Figure 1:**
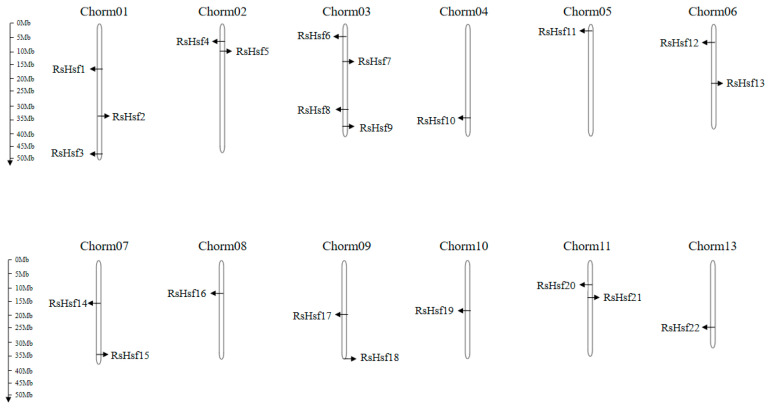
Chromosomal locations of *RsHsfs* in *R. simsii*. The scale represents megabases (Mb) and the sizes of the chromosomes can be determined using the scale given at the left.

Based on the *R. simsii* genome database [26], chromosome mapping of *RsHsfs* showed that the 22 candidate genes were unevenly dispersed on 12 of the 13 chromosomes (all except chromosome 12) (Figure 1). Four genes were located on chromosome 3, three on chromosome 1, two on each of chromosomes 2, 6, 7, 9 and 11, and one on each of chromosomes 4, 5, 8 and 10.

### 2.3. Phylogenetic Classification

**Figure 2:**
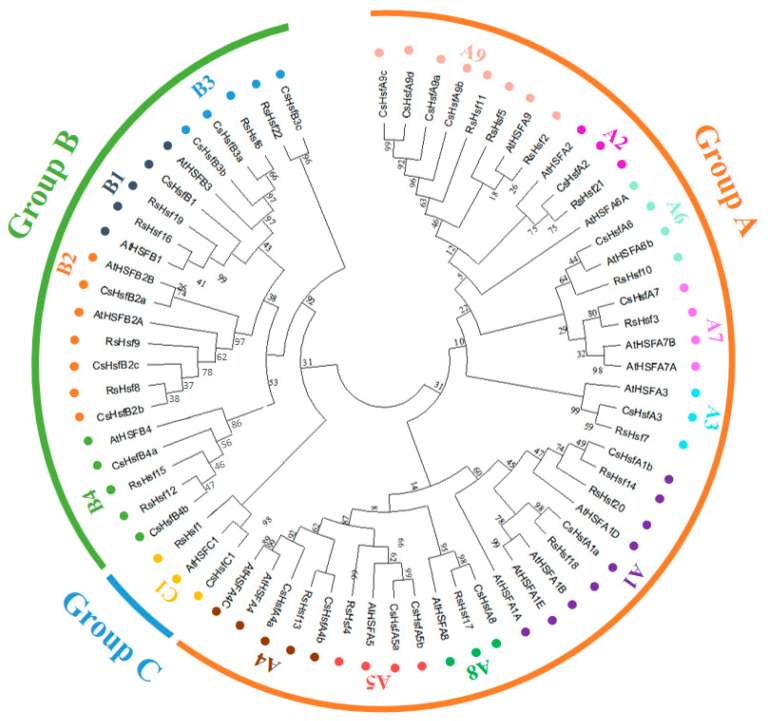
Phylogenetic analysis among the identified Hsf-conserved proteins in *A. thaliana*, *C. sinensis* and *R. Simsii*. The 21 *A. thaliana*, 25 *C. sinensis* and 22 *R. simsii* Hsf sequences were aligned using Muscle. The phylogenetic tree was constructed by MEGA11.0 with the maximum likelihood method, and the bootstrap value was set at 1000 repetitions. Different families and subclasses are indicated by different colors.

To analyze the phylogenetic relationships of RsHsf proteins, we constructed a phylogenetic tree consisting of 22 RsHsf proteins, 25 CsHsf proteins and 21 AtHsf proteins (Figure 2). Based on the well-established classification of AtHsfs in *A. thaliana*, the RsHsfs were divided into three major groups: A, B and C. These groups were further subdivided into 14 subgroups. Group A had the largest number of proteins, and had nine-subgroups A1–A9, with 14 proteins in total. Group B had four subgroups B1–B4, with seven proteins: RsHsf6, RsHsf8, RsHsf9, RsHsf12, RsHsf15, RsHsf16 and RsHsf19. Group C was a separate clade with a single RsHsf member, which was strongly associated with group A.

### 2.4. Gene Structure and Conserved Motifs

**Figure 3:**
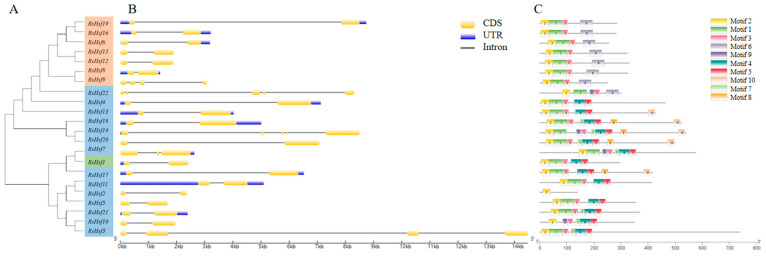
Phylogenetic tree, gene structure, and distribution of conserved motifs. (**A**) Phylogenetic tree constructed using MEGA 11.0 software. (**B**) Schematic of gene structure constructed using tools at gene structure display server. Coding sequences, untranslated regions and introns were represented by yellow boxes, purple boxes and black lines, respectively. The relative position was proportionally displayed based on the kilobase scale at the bottom of the figure. (**C**) Conserved motifs of RsHsf proteins. Each colored box represented a motif in each of the RsHsf proteins, with the motif’ s number represented. The sizes of the gene can be determined using the scale given at the bottom.

The structural diversity of the RsHsf family was analyzed in terms of the exon/intron arrangement of the coding sequences via GSDS (Figure 3) [27]. RsHsfs in the same group typically had similar numbers of introns. Group A members contained one to four introns. All group B members had one introns, except RsHsf14, which had three. The single member of group C had one intron. Among the members of group A, RsHsf14 and RsHsf22 had the largest number of introns (4). RsHsf3 and RsHsf9 each contained three introns, and RsHsf7 contained two introns.

Next, the conserved motifs were predicted. Ten conserved motifs were identified, with lengths ranging from 16 a.a. to 49 a.a, as shown in (Figure S1). All members showed similar motif composition, but there were small differences among the different groups. Among the predicted motifs, motif 1, motif 2 and motif 3 were the most widely distributed and highly conserved. Some motifs were only present in certain groups. For instance, motif 6 was present in all members of group B, but was also present in RsHsf22 in group A. Motif 4 and motif 5 were present in group A and group C, but not in group B. These findings suggested that the structure of RsHsfs is highly conserved, and the complex structure and specific motifs of RsHsf members in different groups may have led to the diversification of protein functions.

### 2.5. Promoter Analysis

**Figure 4:**
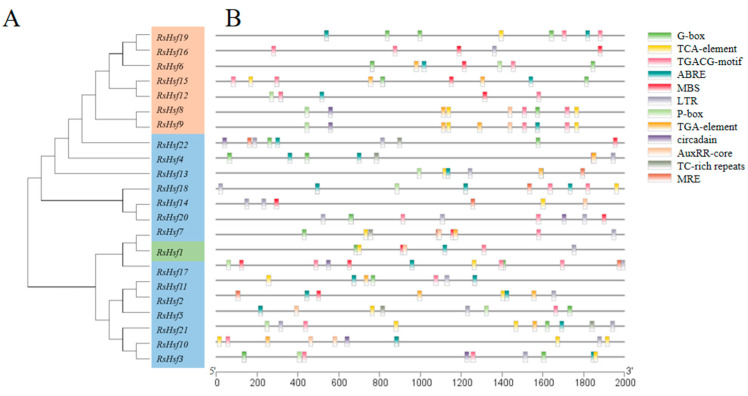
Cis-acting element analysis of promoter regions of RsHsfs. (**A**) Phylogenetic tree constructed using MEGA 11.0 software. (**B**) Cis-acting element analysis of RsHsfs. Different colored boxes were represented by different cis-acting elements. The coordinates at the bottom of the figure indicated the length of the gene promoter, which was defined as 2 kb before the start codon.

To explore the regulatory mode and potential functions of RsHsfs, we extracted the 2-kb promoter region upstream of the initiation codon of each gene and used PlantCARE to search for cis-acting elements (Figure 4). This analysis revealed 12 cis-acting elements in three categories: phytohormone-response elements (ABRE, TCA-element, P-box), stress-response elements (TC-rich repeats, MBS, circadian, LTR) and light-response elements (G-box, MRE). Phytohormone-response elements were most frequently detected, indicating that RsHsfs may be associated with multiple phytohormone signaling pathways. All RsHsf promoter regions contained different types of cis-acting elements, indicating that they play essential roles in growth and development and in stress responses.

### 2.6. Tissue-Specific Transcriptional Profiles of RsHsfs

**Figure 5:**
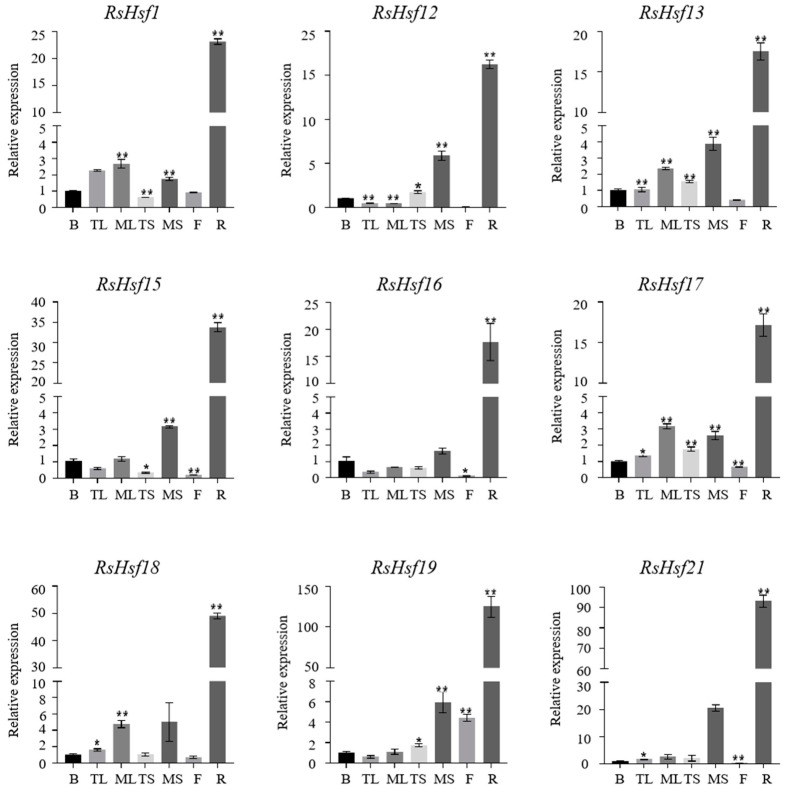
Transcriptional profiles of *RsHsfs* in different tissues. B, buds; TL, tender leaves; ML, mature leaves; TS, tender stems; MS, mature stems; F, flowers; R, roots. Gene expression in buds was regarded as control. Data are mean ± standard deviation (SD), calculated from three biological replicates. Vertical lines represent standard deviation. * and ** indicate significant difference at *p* < 0.05 and *p* < 0.01, respectively.

Gene expression patterns can reflect the potential functions of their encoded products to some extent. Based on the sequences of *RsHsfs*, nine genes were randomly selected for qRT-PCR to analyze their transcriptional profiles in seven different tissues (buds, tender leaves, mature leaves, tender stems, mature stems, flowers, roots) (Figure 5). There were some differences in transcriptional profiles among these genes, indicating that they showed tissue-specific transcriptional patterns. This suggested that *RsHsfs* have a range of functions in physiological and developmental processes. For example, *RsHsf18* transcript levels were relatively high in the mature leaves and mature stems, the transcript level of *RsHsf19* in flowers was significantly higher than those of the other genes, and *RsHsf21* transcripts were detected in mature stems. Interestingly, the nine genes showed similar transcriptional profiles, with the highest transcript levels in the roots and the lowest in the flowers. Because all of the tested genes showed high transcript levels in the roots, we speculated that *RsHsfs* may be closely associated with the regulation of gene expression in the roots.

### 2.7. Transcriptional Profiles of RsHsfs under Abiotic Stresses

**Figure 6:**
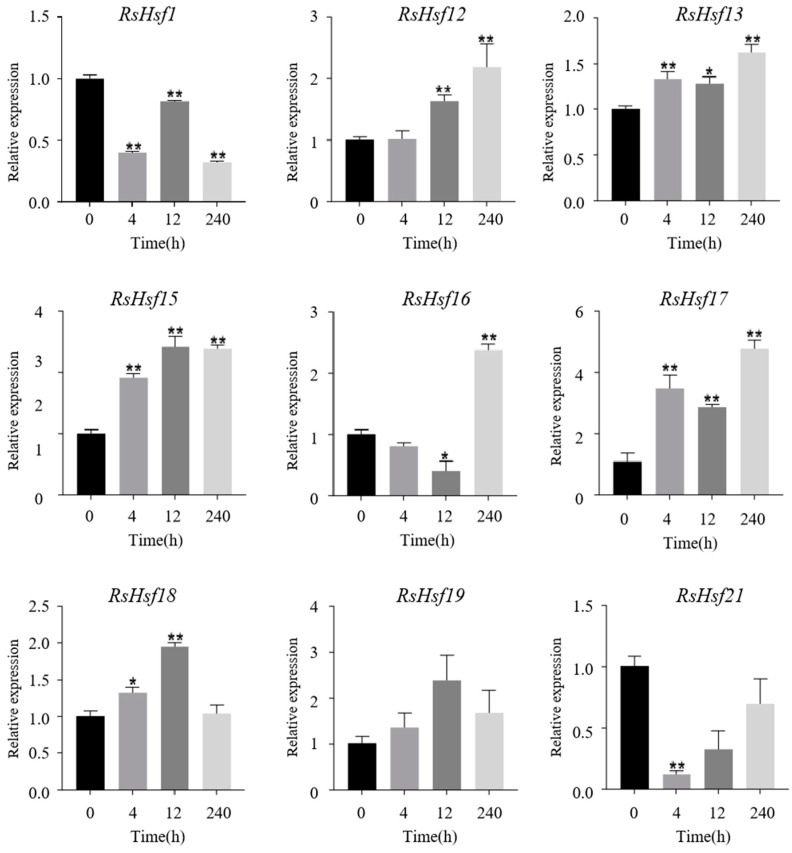
Expression profiles of *RsHsfs* under drought treatment. The detection of transcript levels was performed in leaves. Gene expression at 0 h was normalized to “1”. Data are mean ± standard deviation (SD), calculated from three biological replicates. Vertical lines represent standard deviation. * and ** indicate significant difference at *p* < 0.05 and *p* < 0.01, respectively.

To detect the responses of Hsfs to drought stress, we detected the transcript levels of their encoding genes in *R. simsii* leaves under drought treatment at four time points (0 h, 4 h, 12 h, and 240 h). Most of the genes showed changes in their transcript levels, compared with that at 0 h, but there were differences in the trends in their expression (Figure 6). Under drought stress, *RsHsf1* and *RsHsf21* were down-regulated, whereas the other genes were up-regulated. The transcript levels of *RsHsf12*, *RsHsf13*, *RsHsf15*, *RsHsf16* and *RsHsf17* peaked at 240 h of drought stress, at levels much higher than those at 0 h. Notably, nine out of six genes were up-regulated in the early stage of treatment (4 h and 12 h). The remaining genes were down-regulated in the early stage, but reached peak at 240 h. This suggested that these genes may less sensitive to drought.

**Figure 7:**
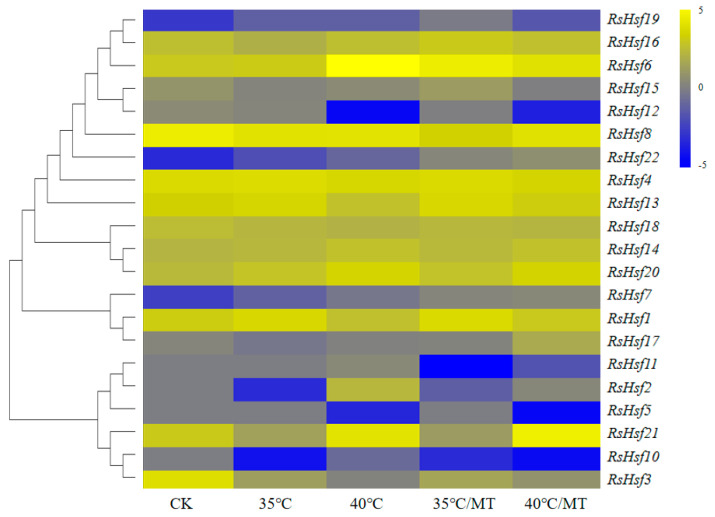
Heat map of the expression profiles of *RsHsf* genes under heat and melatonin treatment. The detection of expressions was performed in leaves. CK, 25 °C melatonin−free plants; 35 °C, 35 °C, melatonin−free plants; 40 °C, 40 °C, melatonin−free plants; 35 °C/MT, 35 °C, melatonin−treated plants; 40 °C/MT, 40 °C, melatonin−treated plants. Log2 transformed FPKM values were used to create heat map. Yellow or blue indicate higher or lower relative abundance or each transcript in each sample.

Transcriptome data were utilized to analyze the transcriptional profiles of *RsHsfs* under heat and melatonin treatments (Figure 7). Most genes showed changes in their transcript levels under these treatments. Under heat stress, nine genes were up-regulated, four were down-regulated, and nine were not expressed or expressed at very low levels. Interestingly, transcripts of four genes, *RsHsf2*, *RsHsf5*, *RsHsf10* and *RsHsf11*, were not detected under normal conditions. However, these genes were strongly regulated under heat stress. Melatonin (N-acetyl-5-methoxypteramine, MT) is an important exogenous growth regulator in plants that mitigates the deleterious effects of various stresses [28,29]. We found that 10 genes were differentially expressed after exogenous application of melatonin under heat stress, compared with their respective transcript levels in melatonin-free rhododendron plants.

### 2.8. Subcellular Localization Analyses

**Figure 8:**
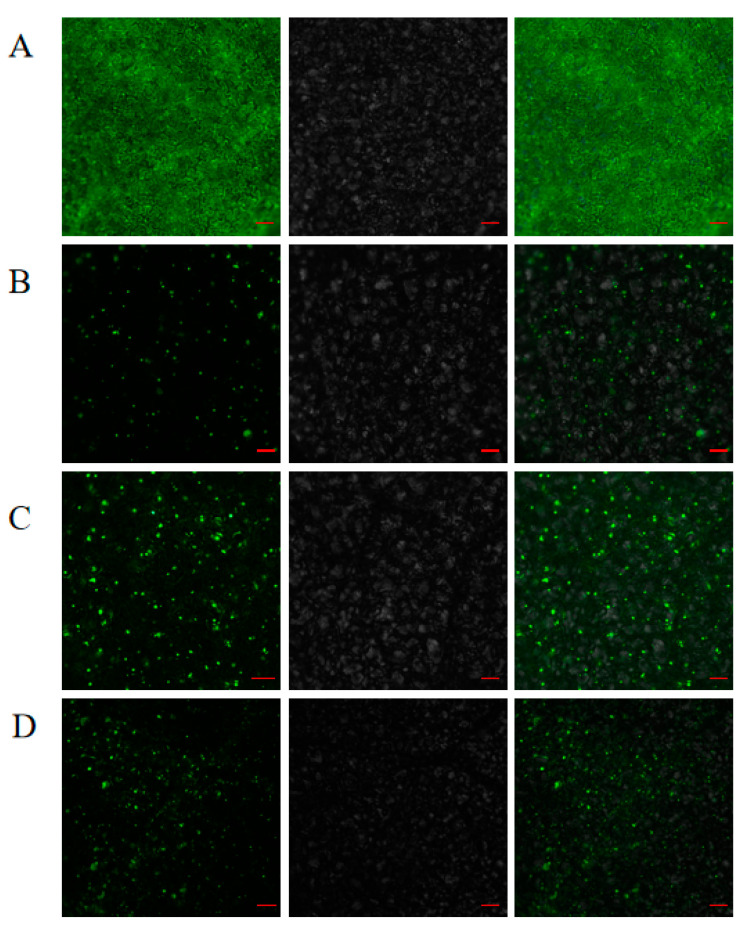
Subcellular localization of RsHsfs. (**A**) 35sGFP. (**B**) RsHsf15-GFP fusion proteins. (**C**) RsHsf16-GFP fusion proteins. (**D**) RsHsf19-GFP fusion proteins. For (**A**–**D**), 35sGFP or RsHsfs-GFP fusion proteins were transiently expressed in tobacco. Left to right: green fluorescence, bright-field and merged microscope images. Scale bars: 100 µm.

To investigate the distribution of *RsHsfs* in cells, three genes (*RsHsf15*, *RsHsf16* and *RsHsf19*) were selected for transient expression analyses (Figure 8). The recombinant plasmids were transiently expressed in tobacco leaves, with 35S-GFP as the control. The 35S-GFP signal was distributed throughout leaves uniformly, while fluorescence signals from the target gene products were presented in the nucleus. This confirmed that *RsHsf15*, *RsHsf16* and *RsHsf19* localized to the nucleus.

## 3. Discussion

Heat shock transcription factors are key transcription factors in plants involved in signaling and response to stress [12]. Analyses of Hsf families have been conducted for more than 20 plant species to date [30]. There are 21 Hsf-encoding genes in *A. thaliana* [8], 24 in tomato [4], 25 in pepper [31], 27 in potato [32], and 25 in *C. sinensis* [33]. The number of Hsf family genes differs widely in different species. To date, no previous studies have identified or functionally characterized Hsfs in *Rhododendron*. In this study, 22 genes were identified in the *R. simsii* genome, similar to the numbers reported in the plants mentioned above. This may be related to the fact that they are dicotyledonous plants with close genetic relationships and conserved evolution. Subcellular localization showed that *RsHsf15*, *RsHsf16* and *RsHsf19* were all located in the nucleus, which was consistent with the reported research results of *TaHsfA1* in wheat [34], *FtHsf18* and *FtHsf19* in Tartary buckwheat [35].

The sequences of plant Hsfs vary greatly, but the basic structure and promoter recognition mode are conserved. Hsfs normally contain five functional domains: a DNA-binding domain (DBD), an oligomerization domain (OD), a nuclear localization signal (NLS), a nuclear export signal (NES), and a C-terminal short activator peptide motif (AHA) [36]. Among these domains, the DBD is usually located at the N-terminal, which is the most conserved region and an essential characteristic of Hsf proteins. The DBD structure is a key identifying character of an Hsf protein. Only proteins with the complete conserved DBD structure are classified as Hsf family members [37].

Based on the differences in DBD and OD domains and their connecting parts, Hsf proteins can be classified into three major groups. In this study, the 22 RsHsfs were divided into three groups and 14 subgroups. Those in the same group were similar, but there were obvious differences among subgroups. In this study, the clustering method of RsHsfs was the same as that of AtHsfs [8]. Group A had the most members, and group C had the fewest. The lack of A7, A10, and C2 subgroups in *R. simsii* indicates that Hsf proteins have a common ancestor, but have constantly evolved in different species. Moreover, our results show that several subfamilies of RsHsf family are larger than that of AtHsf family, including subgroups A2, A5, B1, B4, implying that after the differentiation of these two species, the gene family has expanded more in *R. simsii* than in *A. thaliana* [38].

The signaling pathways in plants form a complex intertwined network, and the same transcription factors can participate in multiple signal transduction events. Cis-acting elements located in gene promoter regions are a crucial part of the signal transduction process, and such elements synergistically regulate gene expression to achieve particular physiological outcomes [39]. In this study, we detected a variety of cis-acting elements in the promoter regions, including ABREs, TCA-elements, and P-box elements. These findings indicate that RsHsfs may be regulated by several kinds of phytohormones. In addition, the presence of MBS, P-box, LTR, and other elements indicates that RsHsfs may be regulated by various abiotic factors. Numerous studies have demonstrated that Hsfs can enhance resistance to stress conditions, such as high temperature [40], salt [41], strong light [42] and oxidation [43]. Interestingly, no HSE elements were detected in these promoter regions, implying that these RsHsfs might not be directly induced by heat stress [27].

Exploration of gene expression patterns can shed light on the biological functions of their encoded products [44]. Therefore, we determined the transcript levels of 9 *RsHsfs* in different tissues. Several *RsHsfs* showed tissue-specific transcript profiles. For example, there were relatively high transcript levels of *RsHsf18* in mature leaves and stems, and of *RsHsf19* in flowers. This result indicated that *Hsfs* are extensively involved in the growth and development of different tissues and organs. Notably, the transcript levels of all *RsHsfs* were higher in the roots than in other tissues, consistent with their expression patterns in other plants. For example, in alfalfa, *MsHsf06* and *MsHsf15* were found to be expressed at higher levels in the roots than in other tissues [45], and the same expression pattern was detected in tea tree [46]. *StHsf19*, *StHsf20*, *StHsf21*, *StHsf22*, *StHsf23* and *StHsf24* of potato [32] were also found to be highly expressed in the roots, buds and tubers of vegetative organs, indicating that their encoded products participate in vegetative growth. In cassava, *MeHsf18* transcript levels were found to be 10–20 times higher in the roots than in the leaves [47]. We speculated that the high transcript levels in roots may be because the roots are the first organ to be affected by changes in soil conditions.

We analyzed the transcript levels of nine genes under drought treatment, and found that seven of them (all except *RsHsf1* and *RsHsf21*) were up-regulated. All these genes had MBS (drought-responsive) elements in their promoter regions. Co-expression of *HaHsfA4a* and *HaHsfA9* genes in tobacco was shown to synergistically enhance the tolerance of transgenic seedlings to drought and oxidative stress [48]. Another study showed that *HsfA1b* controls certain aspects of drought tolerance and water balance in *A. thaliana* [49]. Notably, *RsHsf2*, *RsHsf11* and *RsHsf21* in the A2 subgroup appeared to be strongly induced by heat stress in the present study. *HsfA2* is one of the most important heat shock transcription factors in heat tolerance, and it is expressed only under stress conditions [50]. It accumulates continuously during heat shock and recovery, and it can significantly improve heat resistance both in *A. thaliana* and tomato [51]. In maize, heat stress induces the expression of HsfA2-type genes; *ZmHsf-01*, *ZmHsf-04* and *ZmHsf-17* [10]. In the present study, the transcript levels of *RsHsf2*, *RsHsf13*, *RsHsf17* and *RsHsf18* increased under drought stress, implying that these genes participate in both heat and drought stress responses.

Melatonin is a multifunctional molecule involved in signaling. It is found widely throughout the biosphere. In plants, it has a crucial role in stress resistance and diurnal regulation. Exogenous application of melatonin or accumulation of endogenous melatonin can mitigate damage caused by biotic or abiotic stresses [52], such as high temperature [53], strong light [54], and salt [55]. Recently, we reported that exogenous melatonin can improve the heat tolerance of *Rhododendron* [29]. Photosynthesis is highly sensitive to heat stress. Exogenous melatonin can ameliorate the expression of photosynthetic pathway genes (*RhPGR5A*, *RhATPB*, *RhLHCB3* and *RhRbsA*) in heat-stressed plants. In the present study, application of exogenous melatonin limited the increase in the transcription of several genes under heat stress, including *RsHsfs2*, *RsHsf10* and *RsHsf20*. The differential expression of *RsHsfs* under phytohormone and abiotic conditions highlights their extensive and diverse roles in environmental adaptation.

## 4. Materials and Methods

### 4.1. Plant Materials and Treatments

Two-year-old plants of *R. simsii* and the rhododendron cultivar “FengGuan” growing at Jiyang College of Zhejiang A&F University, Zhejiang, China (29°45′ N, 120°14′ E) were used as the experimental materials in this study. Specifically, “FengGuan” was used for qRT-PCR analyses and *R. simsii* was used for gene cloning experiments. The plants were grown in growth chambers for 2 weeks to adapt to the environmental conditions. The parameters of the growth chambers were set as follows: 14 h light/10 h dark photoperiod, with light supplied at 80 μmol m^−2^ s^−1^, temperature 25 °C day/18 °C night, and 70% relative humidity. Plants of uniform size were treated with heat, drought and melatonin. For the drought treatment, irrigation was withheld for 10 days, and samples were taken at 0 h, 4 h, 12 h and 240 h of treatment. The methods of heat and melatonin treatment were as described in our previous report [29].

### 4.2. Gene Identification and Physicochemical Properties of Putative Proteins

The protein and nucleotide sequences were obtained from *R. simsii* genome database [26]. The conserved DBD domain of Hsf (Pfam: PF00447) was used as the query in a BLASTP search of the *R. simsii* proteome. PFAM and SMART excluded the proteins that did not incorporate DBD domain and HR-A/B domain. Finally, 22 *RsHsf* genes were obtained. ProtParam (http://web.expasy.org/protparam, accessed on 8 June 2023) was used to predict the physicochemical properties of the putative proteins, including the isoelectric point, molecular weight, and number of amino acids. The hydrophilicity and hydrophobicity of proteins were analyzed using Protscale (http://web.expasy.org/protscale/, accessed on 8 June 2023).

### 4.3. Chromosome Location

Information for mapping the 22 *RsHsfs* onto chromosomes was obtained from the annotation file of the *R. simsii* genome database [26]. MG2C (http://mg2c.iask.in/mg2c_v2.0/, accessed on 10 June 2023) was employed to visualize the distribution of *RsHsfs* on 12 chromosomes.

### 4.4. Construction of Phylogenetic Tree

The AtHsfs proteins in *A. thaliana* and CsHsfs proteins in *C. sinensis* were obtained from Phytozome (http://www.phytozome.net/, accessed on 11 June 2023) and Tea Plant Information Archive (TPIA) (http://tpia.teaplant.org/, accessed on 11 June 2023), respectively. These sequences and those of RsHsfs were used to generate a phylogenetic tree with the maximum-likelihood (ML) method using MEGA 11.0 software. The bootstrap method was selected, and the bootstrap repetition was set to 1000. Default values were used for other parameters. Subsequently, the phylogenetic tree was visualized at the iTOL website (https://itol.embl.de/, accessed on 12 June 2023).

### 4.5. Gene Structures and Motifs, and Analysis of Gene Promoter Regions

The gene structures, including intron/exon distribution, were predicted on the website of GSDS (http://gsds.gao-lab.org/, accessed on 15 June 2023). Conserved motifs of RsHsfs were detected and analyzed with MEME (https://meme-suite.org/meme/, accessed on 15 June 2023). The number of motif parameters was set to 10, and other parameters were used with default values. The promoter sequences 2 kb upstream of the initiation codon of RsHsfs were extracted from *R. simsii* genome data, and the cis-acting elements were detected by PlantCare (http://bioinformatics.psb.ugent.be/, accessed on 16 June 2023). The results were visualized using Tbtools software (accessed on 20 June 2023).

### 4.6. Detection of RsHsf Transcript Levels by qRT-PCR

The transcript levels in stressed plants were performed in the leaves of “FengGuan”. Total RNA was isolated and reverse-transcribed using the EASYspin Plus Complex Plant RNA Kit and TRUEscript RT Master Mix (Aidlab, Beijing, China) according to the manufacturer’s protocols. The obtained RNA and cDNA products were kept at −80 °C until use. Primer Premier5 was used to design gene-specific primers, which were synthesized by Tsingke (Beijing, China) (Table S1). In the qRT-PCRanalyses, the 10 µL reaction mixture consisted of 5 µL MonAmpTM SYBR^®^ Green qPCR Mix, 3 µL cDNA, 0.5 µL Primer-R, 0.5 µL Primer-F, and 1 µL ddH_2_O. The PCR cycling program was as follows: 10 min at 95 °C, 40 cycles of 15 s at 95 °C, 15 s at 59 °C, and 10 s at 72 °C. The glyceraldehyde 3-phosphate dehydrogenase (*GAPDH*) gene was used as an internal control [56], the Ct values were calculated on the Roche LightCycler 480 II instrument (Roche Diagnostics, Germany) automatically. The gene transcript levels were calculated using the 2^−△△CT^ method [57]. Data were analyzed via two-tailed Student’ s *t*-test with *p* < 0.05 (*) and *p* < 0.01 (**) set as the thresholds for determining significance. Charts were constructed using Graphpad prism 9.0. Three independent biological replicates were analyzed for each sample.

### 4.7. Subcellular Localization Analyses

The coding sequences of *RsHsfs* without the stop codon were amplified from the cDNA of *R. simsii*. Each product was ligated into the binary vector (pCAMBIA1300-35S::GFP) to produce a Pro35S::RsHsf::GFP construct, which was then transformed into *Agrobacterium tumefaciens* strain EHA105. The transformed *A. tumefaciens* was used to infiltrate the leaves of 4-week-old tobacco plants [58]. At 48–72 h after infiltration, GFP signals were observed using Nikon Eclipse Ni-U microscope (Nikon, Tokyo, Japan).

## 5. Conclusions

In this study, 22 *RsHsf* genes were identified from genomic data of *R. simsii* for the first time. The gene structures, phylogenetic relationships and conserved motifs of RsHsf family members were determined. Transcriptional profile analyses revealed that *RsHsfs* display significant specificity of expression in different tissues, and play important roles in responses to abiotic stress. The results of this study provide basic information about *RsHsfs*, and give new insights into the function of *Hsf* genes in abiotic stress resistance in *R. simsii*.

## Figures and Tables

**Table 1:** Physicochemical properties of putative RsHsfs.

Gene Name	Gene ID	Amino Acid	Molecular Weight (KDa)	Theoretical Isoelectric Point	Instability Index	Grand Average of Hydropathicity
*RsHsf1*	Rhsim01G0094400	296	33.01	5.42	57.20	−0.47
*RsHsf2*	Rhsim01G0168300	140	16.63	4.50	55.62	−0.37
*RsHsf3*	Rhsim01G0272100	740	83.56	6.84	53.50	−0.74
*RsHsf4*	Rhsim02G0062200	464	52.26	5.35	60.59	−0.82
*RsHsf5*	Rhsim02G0071200	354	39.70	6.29	44.08	−0.70
*RsHsf6*	Rhsim03G0027200	257	29.01	5.56	70.97	−0.84
*RsHsf7*	Rhsim03G0081300	577	63.55	4.70	60.67	−0.52
*RsHsf8*	Rhsim03G0178900	327	36.11	5.45	54.93	−0.64
*RsHsf9*	Rhsim03G0236900	250	28.23	9.45	46.61	−0.70
*RsHsf10*	Rhsim04G0197300	352	39.11	4.83	56.31	−0.61
*RsHsf11*	Rhsim05G0018200	415	46.58	4.83	51.85	−0.72
*RsHsf12*	Rhsim06G0051500	332	37.54	6.73	61.12	−0.61
*RsHsf13*	Rhsim06G0124200	428	48.77	5.26	56.09	−0.77
*RsHsf14*	Rhsim07G0130600	540	59.94	5.16	59.15	−0.60
*RsHsf15*	Rhsim07G0227900	324	36.93	5.94	58.81	−0.68
*RsHsf16*	Rhsim08G0100600	282	31.20	6.38	33.73	−0.77
*RsHsf17*	Rhsim09G0088300	417	47.75	4.64	50.57	−0.51
*RsHsf18*	Rhsim09G0213400	524	57.63	5.50	52.32	−0.43
*RsHsf19*	Rhsim10G0118300	286	31.88	8.76	31.74	−0.74
*RsHsf20*	Rhsim11G0019200	501	55.43	4.86	65.34	−0.63
*RsHsf21*	Rhsim11G0043100	370	42.03	4.79	67.84	−0.54
*RsHsf22*	Rhsim13G0177200	300	35.04	9.13	46.37	−0.79

## Data Availability

The data presented in this study are available on request from the corresponding author. The data are not publicly available due to privacy.

## Supplementary Materials

The following supporting information can be downloaded at: https://www.mdpi.com/article/10.3390/plants12223917/s1, Figure S1: The specific sequence information of 10 motifs. Table S1: Primers of the RsHsfs for qRT-PCR.
