# Supplementary material for: A Genome-Wide Analysis and Expression Profile of Heat Shock Transcription Factor (Hsf) Gene Family in Rhododendron simsii
Source: Plants (Basel). 2023 Nov 20;12(22):3917. doi: 10.3390/plants12223917 (PMC10674592; doi:10.3390/plants12223917)
Supplement: Supplementary file 1 [file plants-12-03917-s001.zip › plants-2626705-supplementary.pdf]

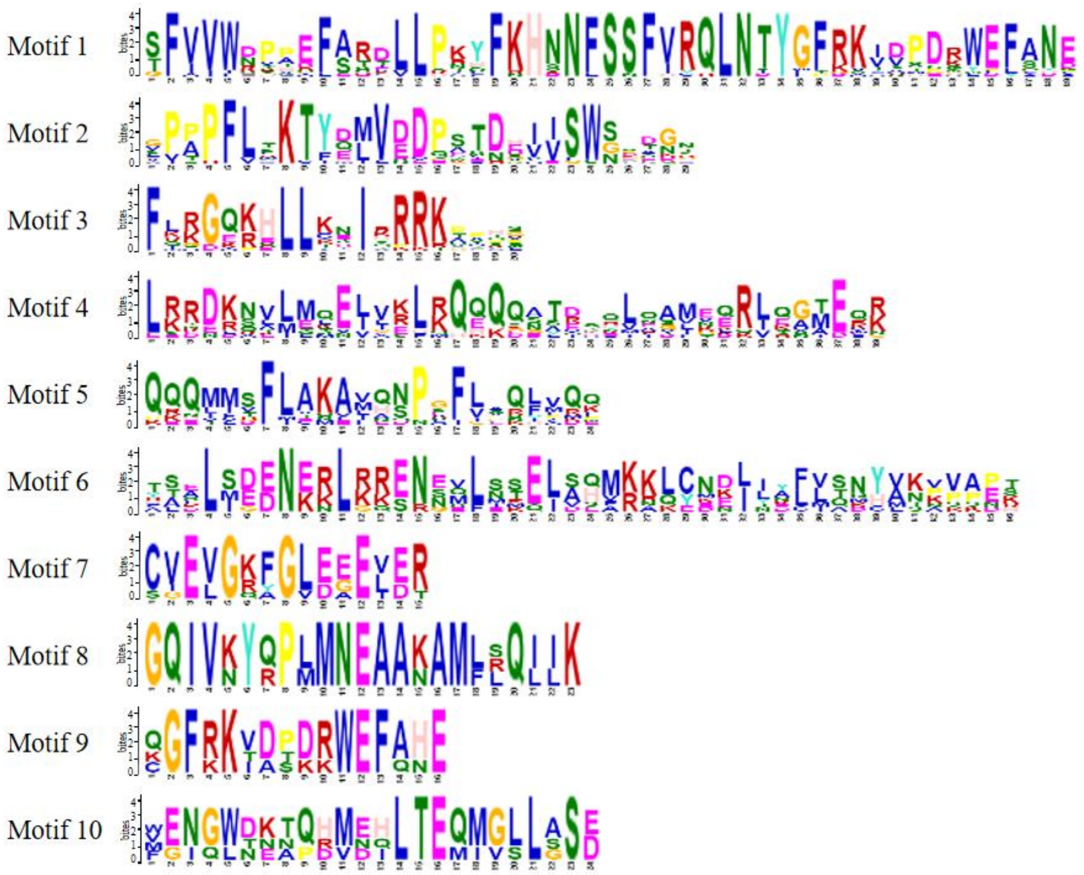

Figure S1. The specific sequence information of 10 motifs. The letters represent amino acids, the color does not represent any meaning, which are automatically synthesized by the MEME, and the height of the letters represents the motifs conservation.

Table S1. Primers of the RsHsfs for qRT-PCR

Primer	Sequence
qRT-RsHsf1-F	ACGGTTTCCGAAAGGTCGAT
qRT-RsHsf1-R	TGACCTTGCTGTGCTTCCTT
qRT-RsHsf12-F	GCTTTCGGAGGACAACGAGA
qRT-RsHsf12-R	AGGCGGCGGCAAATTAGATA
qRT-RsHsf13-F	CTTCCACTGCCTTCATCCGT
qRT-RsHsf13-R	TGGAGGGCAATAGACGATGC
qRT-RsHsf15-F	TACAACACCACCACCACCAC
qRT-RsHsf15-R	GTTTCCTCATGTGCGCGATT
qRT-RsHsf16-F	TCAAGCGAGGACAACGAGAG
qRT-RsHsf16-R	TCGGCGTTCTTGGATGGTAG
qRT-RsHsf17-F	GGCTATGCAAAGTCCTGGGT
qRT-RsHsf17-R	ATTGGCGGCTGATACCTCAC
qRT-RsHsf18-F	CCGCAACGATGATTCCAACC
qRT-RsHsf18-R	CACGTTCCAGACAGCAAAGC
qRT-RsHsf19-F	AGGGAGCTCCTCACACAGAT
qRT-RsHsf19-R	GGCGTTCTTCGAGTCTAGCA
qRT-RsHsf21-F	GAGGGCAGAAACACCTCCTG
qRT-RsHsf21-R	TTCTGCTGGTGCTGTCTGAG
